# Aberration in the Appreciation of Colours

**Published:** 1844-05-18

**Authors:** 


### ABERRATION IN THE APPRECIATION OF COLOURS.

Dr. Boys de Loury published the following case in the *Revue Medicale*:—Mr. H—— was obliged to give up his business as a dyer on account of his being incapable of distinguishing the different colours. The orange was yellow, a bit of brown and orange-coloured silk was of a uniform darker yellow, apricot was yellow, lilac was blue, violet was grey; garance, crimson, vermillion, were all violet; rose was a dirty white, brown was black. The author, after passing in review the different causes, thinks that it ought to be attributed to an atrophy of the retina.—*Ibid.*

